# Case report: Disseminated aspergillosis complicating influenza

**DOI:** 10.1016/j.mmcr.2019.04.007

**Published:** 2019-04-27

**Authors:** Shanan Immel, Edwin Yu

**Affiliations:** a University of Arizona College of Medicine Phoenix, 550 E Van Buren St, Phoenix, AZ 85004, USA; b Banner University Medical Center Phoenix, 1111 E McDowell Rd, Phoenix, AZ 85006, USA

**Keywords:** Aspergillus, Influenza, DKA, Disseminated, Immunocompetent

## Abstract

Aspergillus can cause a wide variety of diseases ranging from hypersensitivity diseases to invasive infection. Invasive disease most commonly occurs in severely immunocompromised patients such as chemotherapy-induced neutropenia and transplantation. It is a less well-recognized complication in critically ill patients without traditional risk factors. We describe a case of invasive aspergillosis complicating influenza and diabetic ketoacidosis that disseminated to the central nervous system and led to demise despite high-intensity antifungal therapy.

## 1 Introduction

*Aspergillus* is a common soil and environmental fungus that can cause a variety of different diseases in both immunocompromised and immunocompetent hosts. Invasive aspergillosis, which is the most dreaded form of the disease, typically occurs in individuals with profound immunosuppression such as hematopoietic stem cell transplant, solid organ transplant, chemotherapy-induced neutropenia, and significant corticosteroid exposure [1]. A less common but well-recognized risk factor for invasive pulmonary aspergillosis (IPA) is critical illness requiring mechanical ventilation. It is estimated that the incidence of IPA in the ICU population may be as high as 7%, where patients commonly have multiple risk factors including acute respiratory failure, mechanical ventilation, steroid use, acute renal failure and comorbid illness [2]. Invasive aspergillosis most commonly presents as sinopulmonary disease due to inhalation of spores. From the lungs, Aspergillus may disseminate hematogenously to the skin, bone, visceral organs and the central nervous system.

Other presentations of *Aspergillus* disease primarily depend on the pulmonary structure or underlying disease of the patient. In patients who have a pre-existing cavitary lesion in their lungs, *Aspergillus* can sometimes colonize that space and form an Aspergilloma or fungus ball. This may be the most common disease caused by Aspergillus species, although fungus balls can sometimes be caused by other fungi as well*.* Aspergilloma is a limited disease and typically does not invade into the lung parenchyma or blood vessels [3]. Aspergilloma can be seen on computed tomography and confirmed by culture or histological identification of Aspergillus in sputum, bronchoalveolar lavage fluid, or trans-thoracic needle aspirations [4]. Another disease entity caused by Aspergillus is Allergic Bronchopulmonary Aspergillosis (ABPA), which is a hypersensitivity reaction, with the majority of cases in those with cystic fibrosis or asthma, especially those who are corticosteroid-dependent [3]. ABPA may manifest as developing chronic and intermittent lung inflammation sometimes leading to bronchiectasis. Diagnosis is clinical and requires a number of criteria to make ABPA likely: asthma, peripheral eosinophilia, skin test reactivity to Aspergillus, precipitating IgG antibodies to Aspergillus, and elevated serum IgE [4]. Aspergillus may also present in a number of overlapping chronic manifestations. Chronic disease almost always occurs in the setting of previous structural lung disease and manifestations are probably modulated by varying and ongoing immunosuppression. Under the umbrella of chronic pulmonary aspergillosis (CPA), there are a couple of entities of note. In those with some degree of immunosuppression (chronic corticosteroid-use, AIDS, diabetes mellitus, alcoholism) patients may develop a more rapidly evolving form of disease called chronic necrotizing pulmonary aspergillosis (CNPA) characterized by the formation of nodules, consolidations with or without the development of cavities in the lungs. A less destructive and slowly evolving form of disease characterized by single or multiple cavities with thick walls is called chronic cavitary pulmonary aspergillosis (CCPA). In some patients with CCPA who develop extensive fibrosis, they may be classified as chronic fibrosing pulmonary aspergillosis [1]. Isolated tracheobronchial disease is a rare subtype of Aspergillus and may be diagnosed as a bronchial plaque and can present with or without parenchymal disease [1].

## 2 Case

**Fig. 1:**
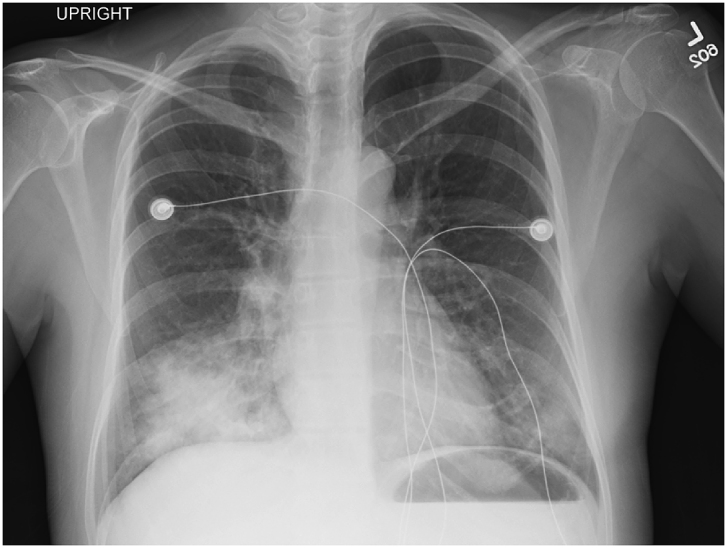
Chest x-ray on hospital day 1 showing right middle and left lower lobe consolidation.

A 38-year-old woman with uncontrolled type 2 diabetes presented to the emergency room with 1 week of chills, fever, nausea, vomiting, cough and sore throat. In the emergency room, her initial exam demonstrated bilateral rales as well as mild distress. Skin, abdominal, and cardiac exams were normal. Her initial vital signs showed that she was afebrile, tachycardic to the 130s, tachypneic with a respiratory rate of 37, and O_2_ saturation of 98% on room air. Her initial work-up was significant for WBCs of 11,600/mm^3^ and glucose of 776 mg/dL with an anion gap of 20. On venous blood gas pH was 6.84 and HCO3 of 4 mmHg. Her initial chest x-ray showed a right middle and left lower lobe consolidation compatible with pneumonia (Fig. 1).

**Fig. 2:**
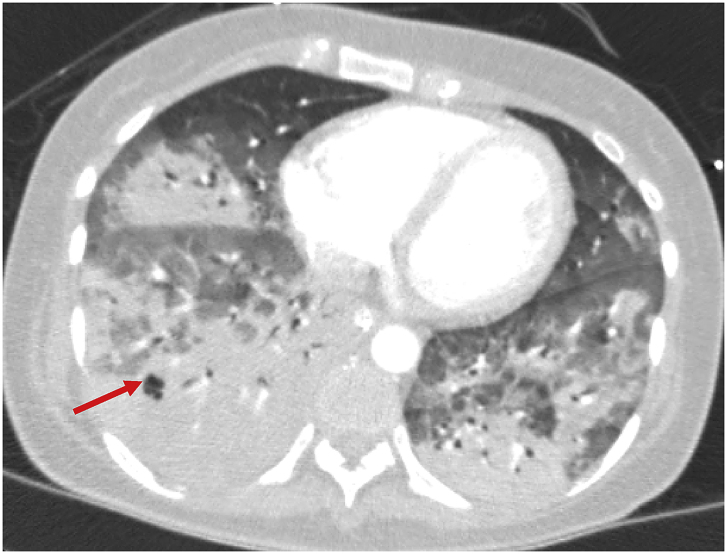
CT Chest on HD 5 showing multifocal nodular consolidations with early cavitation (red arrow).

She developed respiratory distress and altered mental status while undergoing evaluation in the ED and required intubation and admission to the ICU for acute respiratory failure, septic shock, and diabetic ketoacidosis. She was started on broad-spectrum antibacterials as well as oseltamivir. A nasopharyngeal swab was positive for influenza B and blood cultures were positive for *Streptococcus pneumoniae*. A bronchoscopy performed 14 hours after admission demonstrated many Gram-positive cocci in pairs and whitish plaques in the tracheobronchial tree which were overlooked. BAL cultures confirmed *Streptococcus pneumoniae* infection. On hospital day 4 due to continued alteration in mental status and persistent fevers and leukocytosis she underwent Head CT and LP which were unremarkable. Due to lack of clinical improvement she underwent CT chest on hospital day 6 demonstrating multifocal nodular consolidations with early cavitation (Fig. 2).

**Fig. 3:**
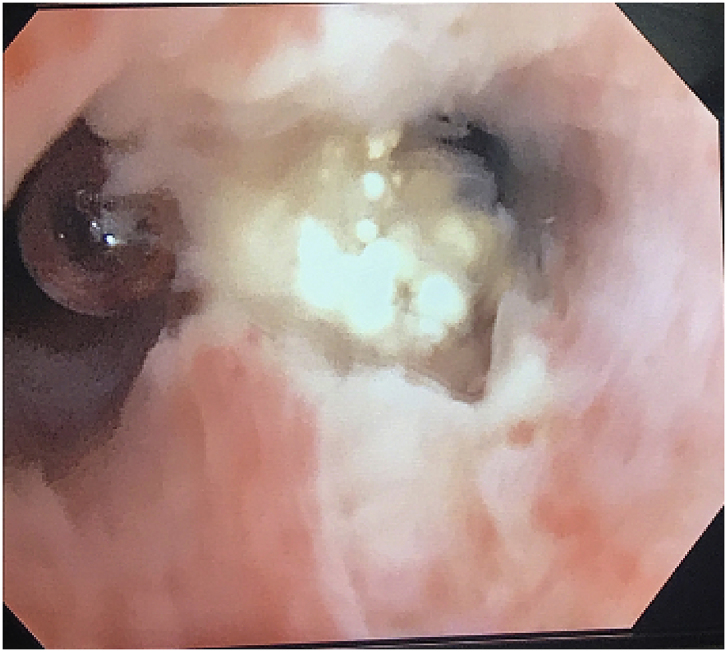
Bronchoscopy on HD 9 showing white fungus ball in left main bronchus.

On hospital day 9 *Aspergillus fumigatus* was finally identified on her admission BAL. She was immediately started on voriconazole. Further diagnostic evaluation included serum Fungitell ((1–3)-ß-D-Glucan) and Aspergillus antigen which were strongly positive. Repeat bronchoscopy showed a whitish collection of material in the left main bronchus (Fig. 3) which was biopsied and revealed abundant fungal hyphae on pathology consistent with Aspergillus.

**Fig. 4:**
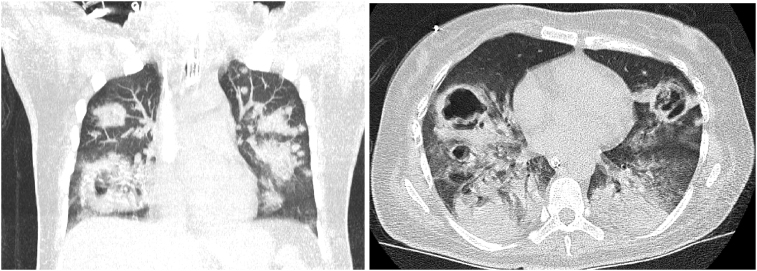
CT Chest on HD11. Left: coronal. Right: axial. Showing new large areas of cavitation bilaterally with some surrounding groundglass halos, consistent with necrotizing pneumonia, possibly invasive aspergillus.

On hospital day 11 a repeat CT chest showed new large areas of cavitation bilaterally with some surrounding groundglass halos, mostly at sites of previous consolidations (Fig. 4).

Due to worsening cavitary disease on imaging and the burden of infection micafungin was added on hospital day 12. On hospital day 13 an MRI brain with and without contrast was ordered due to the patient's continued lack of neurological improvement which showed multifocal area of prominent T2 hyperintensity in both frontal lobes and left thalamus suggestive of CNS aspergillosis. Due to the presence of tracheobronchial aspergillosis and evidence of endobronchial spread on imaging, nebulized amphotericin B was added.

**Fig. 5:**
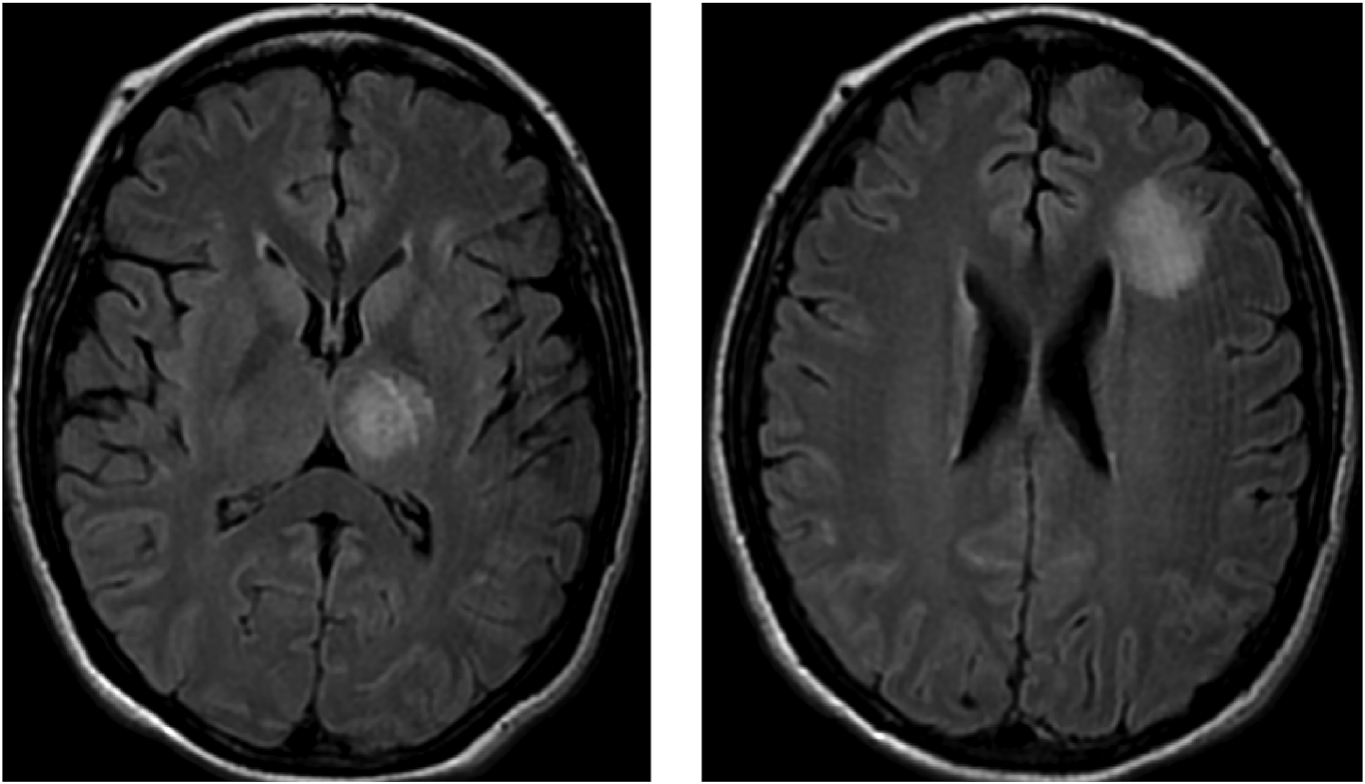
Brain MRI on HD 20. Left: thalamic lesion. Right: frontal lobe lesion.

On hospital day 15, bronchoscopy showed exudate throughout the tracheobronchial tree. Cryotherapy was performed in the left upper lobe for debulking of fungal burden. For five more days she was continued on high-intensity antifungal treatment without neurological improvement. On HD 20 amphotericin B was switched from nebulized to intravenous. A repeat MRI brain on HD 20 showed unresolved lesions in the frontal lobe and some scattered 5mm foci in the left corona radiata while showing a smaller yet still present thalamic lesion (Fig. 5). The patient was kept on her treatment but did not have further clinical improvement. After some additional radiographic evidence of bony involvement in some of her ribs, and due to a lack of response despite antifungal treatment, she was diagnosed with overwhelming disseminated aspergillosis and her family agreed to withdraw care. On HD 25 she was extubated and expired the same day.

## 3 Discussion

There is growing recognition of invasive pulmonary aspergillosis complicating severe influenza infections since the H1N1 2009 epidemic [5]. One proposed mechanism for how influenza puts the lungs at increased risk of invasive aspergillosis is pathological evidence for the virus causing diffuse alveolar damage, necrosis of bronchial mucosa, and submucosal hemorrhage. Influenza viruses and strains infect the epithelium from the nasal passage to the bronchioles, and some high virulence strains may cause more diffuse damage. This damage to epithelium is why classically one may see post-influenza bacterial pneumonia [6] as seen in this case report. Another theory for post-influenza infections is a systemic viral-induced Th1/Th2 changes and lymphopenia which puts patients at risk for secondary bacterial and occasional fungal infection [7]. Since the immune control of Aspergillus is dependent on T-cell immunity, the viral-induced lymphopenia caused by an influenza infection may be the key to increased risk for secondary aspergillus infections [8]. This damage may also disrupt the epithelium and allow for fungal hyphae invasion and hematogenous spread in severe cases. Invasive Aspergillus infections are more commonly associated with influenza A, although a smaller number cases in the literature are associated with influenza B, which can also cause ciliary stripping and focal necrosis and hemorrhage in the upper respiratory tract [9].

In this case report, the patient had a superimposed pneumococcal pneumonia complicating her influenza, which made detection of *Aspergillus* challenging. The influenza infection could have made her more susceptible to both secondary infections, but the bacterial one was discovered and treated first, while the fungal infection was not discovered until much later in her hospital course. Since clinical and radiological manifestations of aspergillosis may be non-specific, it is critical that there is prompt recognition of this fungal infection so antifungal therapy can be initiated early [4]. Based on reported cases, invasive aspergillosis complicating influenza appears to occur early with the median time of 5–6 days between admission and diagnosis of aspergillosis [5,7].

Another peculiar aspect of this case was that the patient presented on admission with evidence of fungal disease, as the initial bronchoscopy eventually grew Aspergillus. Intensive care and intubation are associated with invasive Aspergillus infections, with patients developing disease after multiple days already in the ICU suggesting nosocomial acquisition [2,10]. This patient was intubated in the ED and transferred to the ICU and was not diagnosed with Aspergillus until hospital day 9, but she had evidence of the disease upon admission consistent with community-acquired infection. Notably, she presented with tracheobronchial involvement with bronchial plaques noted on her admission bronchoscopy. Invasive tracheobronchial aspergillosis is a rare sub-type of invasive aspergillosis limited to the tracheobronchial tree and makes up only about 7% of pulmonary cases and can present as ulcerative or pseudomembranous forms [14]. It is usually seen in severely immunocompromised patients and in those who have received a lung transplant. It has been proposed that Aspergillus tracheobronchitis may also be occasionally present in more mildly immunocompromised patients such as the elderly, patients with COPD, post-influenza, or uncontrolled diabetes [13].

Only one form of Aspergillus, Chronic Necrotizing Pulmonary Aspergillosis (CNPA), has been associated with diabetes in the literature. CNPA can present with constitutional symptoms and dyspnea and may appear radiologically indistinguishable from an aspergilloma. While a simple aspergilloma has a thin-walled cyst, complex aspergillomas may present with thick-walled cavities and parenchymal lung infiltrates. CNPA may present as a complex aspergilloma that begins as a small or ill-defined area of consolidation that progresses to well-defined cavities. Expanding cavities and paracavitary invasion is typical [11]. Patients with CNPA may manifest a more rapidly evolving disease [1]. Additionally, diabetics are also known to have impaired host responses, which could indicate that uncontrolled diabetes is a non-traditional risk factor for invasive Aspergillus infections as well [13].

Cerebral dissemination has the worst prognosis of any of the many manifestations of aspergillosis, with mortality exceeding 90%. Voriconazole can be used due to good CNS penetration [12]. A retrospective study showed that in cases of cerebral aspergillosis treated with voriconazole 35% had a complete or partial response, but the overall survival rate was still 31% [4,11].

The use of combination high-intensity antifungal treatment for the treatment of invasive Aspergillus infection has not been extensively studied. Voriconazole remains first-line therapy for Aspergillus infections but when infections prove refractory, antifungals from different classes can be added or can replace the initial regimen. Likewise, the management of invasive tracheobronchial aspergillosis primarily entails the use of systemic antifungal therapy. Adjunctive treatment measures can include inhaled amphotericin and bronchoscopic debridement in select cases [14].

This case illustrates a number of interesting features of *Aspergillus* infections. It can be challenging to diagnose early, especially in patients who do not have traditional immunosuppressive risk factors. The lack of specific symptoms and early radiological findings also make the diagnosis of invasive infection difficult. Radiographically it can be challenging to distinguish between early pulmonary Aspergillus and bacterial pneumonia, especially if the patient has not had prior cavitary lesions and is not showing classic radiological signs of Aspergillus. Tracheobronchial involvement can be seen in post-influenza invasive aspergillosis and should be recognized at bronchoscopy which may be critical for early diagnosis. Most importantly, early diagnosis and initiation of treatment remain critical to limit progression of disease and improve the survivability of invasive aspergillosis.

## Conflict of interest

The authors declare that there are no conflicts of interest regarding the publication of this paper.
